# The Colombian Strain of *Trypanosoma cruzi* Induces a Proinflammatory Profile, Neuronal Death, and Collagen Deposition in the Intestine of C57BL/6 Mice Both during the Acute and Early Chronic Phase

**DOI:** 10.1155/2022/7641357

**Published:** 2022-01-12

**Authors:** José Rodrigues do Carmo Neto, Arthur Wilson Florêncio da Costa, Yarlla Loyane Lira Braga, Fernanda Hélia Lucio, Ana Luisa Monteiro dos Santos Martins, Marlene Antônia dos Reis, Flávia Aparecida de Oliveira, Mara Rúbia Nunes Celes, Marcos Vinicius da Silva, Milton Adriano Pelli Oliveira, Juliana Reis Machado

**Affiliations:** ^1^Department of Bioscience and Technology, Institute of Tropical Pathology and Public Health, Federal University of Goiás, 74605-450 Goiania, GO, Brazil; ^2^Discipline of General Pathology, Institute of Biological and Natural Sciences of Federal University of Triângulo Mineiro, 38025-180 Uberaba, Minas Gerais, Brazil; ^3^Department of Microbiology, Immunology, And Parasitology, Institute of Biological and Natural Sciences of Federal University of Triângulo Mineiro, 38025-180 Uberaba, Minas Gerais, Brazil

## Abstract

The objective of this study was to evaluate the histopathological changes caused by infection with the Colombian strain of *Trypanosoma cruzi (T. cruzi)* in the acute and chronic experimental phases. C57Bl/6 mice were infected with 1000 trypomastigote forms of the Colombian strain of *T. cruzi*. After 30 days (acute phase) and 90 days (early chronic phase) of infection, the animals were euthanized, and the colon was collected and divided into two parts: proximal and distal. The distal portion was used for histopathological analysis, whereas the proximal portion was used for quantification of pro- and anti-inflammatory cytokines. In addition, the weight of the animals and parasitemia were assessed. The infection induced gradual weight loss in the animals. In addition, the infection induced an increase in interferon gamma (IFN*γ*) and tumor necrosis factor-alpha (TNF-*α*) in the intestine in the acute phase, in which this increase continued until the early chronic phase. The same was observed in relation to the presence of intestinal inflammatory infiltrates. In relation to interleukin (IL)-10, there was an increase only in the early chronic phase. The Colombian strain infection was also able to induce neuronal loss in the myenteric plexus and deposition of the collagen fibers during the acute phase. The Colombian strain of *T. cruzi* is capable of causing histopathological changes in the intestine of infected mice, especially in inducing neuronal destructions. Thus, this strain can also be used to study the intestinal form of Chagas disease in experimental models.

## 1. Introduction

More than 100 years after the discovery of the etiologic agent of Chagas disease (CD), *Trypanosoma cruzi*, the disease still has a great socioeconomic impact. This disease is estimated to cost approximately $627 million per year for global public health [1]. In addition, due mainly to the loss of productivity and premature death of those infected, approximately 1.2 billion dollars are spent annually worldwide [2]. The main pathological manifestations of CD include the heart, digestive system, and chagasic megacolon, which accounts for 10–20% of cases that evolve the digestive forms [3]. Little is known about the mechanisms involved in progression, and because of this, experimental models are used to assist in the search for answers.

The strain of *T. cruzi* most commonly used in experimental models of mouse [4, 5], rat [6, 7], or dog [8] that mimic the intestinal form of CD is the Y strain. Thus, most of the findings related to intestinal histopathological and immunological changes due to *T. cruzi* infection are related to this strain. Mice of the C57BL/6 strain infected with the Y strain, for example, show changes in the width of the colon and thickness of the muscle layer and an increase in the inflammatory infiltrate in the intestine, as well as tissue parasitism, myositis, ganglionitis, and periganglionitis during the acute experimental phase [9]. During the chronic phase, there is an increase in the deposition of collagen fibers in the intestine, which is associated with fibrosis in the organ [10]. The same process has also been reported in the human chagasic megacolon [11]. In addition, infection by this strain leads to neuronal decrease in experimental models in both the acute and chronic phases [5, 12], which is also a milestone in the progression of the megacolon in humans [13, 14].

There is a diversity of strains of *T. cruzi* that have different biological behaviors, mainly related to molecular biology, tissue tropism, and the form of the developed DC [15, 16]. While infection in experimental models with strains such as Y [5, 10] and MORC-1 [17] causes intestinal neuronal destruction, strains such as Ninoa, Queretaro [18], and Brazil [19], which induce intestinal changes, have not been evaluated for the number of neurons in the intestinal plexuses.

Furthermore, the Colombian strain is widely used for studies based on experimental Chagas heart disease all because of the myotropism, mainly cardiac and skeletal, that this strain has [20, 21]. The presence of the parasite and intestinal changes has been reported, although less frequently, in infections with this strain [4, 22]. However, in these studies, the relationship between intestinal neuronal number, cytokine behavior, and fibrose deposition has not been evaluated in mice. Thus, the objective of this study was to assess whether the Colombian strain is related to immunopathological changes and neuronal destruction in the intestine, both during the acute and early chronic phases of experimental infection.

## 2. Material and Methods

### 2.1. Animals, Infection, and Euthanasia

The study was approved by the Ethics Committee on the Use of Animals of the Federal University of Goiás (protocol number: 051/19). Thus, all conditions of handling, maintenance, and euthanasia of the animals were followed as indicated.

The animals used in this study were bred and donated by the Bioterium of the Institute of Tropical Pathology and Public Health of the Federal University of Goias. Male C57Bl/6 mice (22–27 g) were infected, subcutaneously, or not with 1000 trypomastigote forms of *T. cruzi* Colombian strain obtained from BALB/c mice at the peak of parasitemia. From the day of infection, the animals were followed for 30 days (*n* = 5) and 90 days (*n* = 4) during acute and early chronic phases, respectively. Control animals without infection were also followed for 30 days (*n* = 5) or 90 days (*n* = 5). At the time of euthanasia (cervical dislocation after confirmation of the anesthetic status, induced by 50 mg/kg of xylazine hydrochloride intraperitoneally), the final portion of the colon was collected, and the proximal portion was used for the measurement of cytokines, whereas the distal portion was used for histopathological analysis.

### 2.2. Parasitemia and Animal Weight

Parasitemia of infected mice was performed at 3-day intervals until the total disappearance of blood trypomastigotes. For this, 5 *μ*L of blood was collected from the tail vein of the animals and then placed on a slide and cover slip. Then, 50 random fields were evaluated under an ordinary light microscope to count the circulating trypomastigotes. The weight of the animals was collected on the day of infection (0 day) and on the subsequent days for euthanasia (30 and 90 days).

### 2.3. Histopathological Evaluations

The distal part of the colon of the animals was washed with ×1 phosphate buffered saline (PBS), transferred to a filter paper, and fixed within 48 h with 4% paraformaldehyde. The fixed material was then processed according to a previous study [18].

For analysis of the inflammatory infiltrate, three serial cuts (100 *μ*m apart) were stained with hematoxylin-eosin. Then, 10 photos of each cut (final = 30 photos), under ×400 magnification, were captured using a common light microscope attached to the camera. First, the intensity of the inflammatory infiltrate was established qualitatively in the submucosa and muscle, following the classification of 1 for mild, 2 for moderate, and 3 for accentuated. After this classification, the average of the 30 photos was obtained and classified according to the following score: 0–0.3, normal; 0.4–1.0, discrete; 1.1–2.0, moderate; and 2.1–3.0, accentuated [4].

Slides stained with Giemsa stain were used to quantify the intestinal nerve ganglia. Four serial slices, with 100 *μ*m between each slice, were evaluated under standard optical microscopy at ×400 magnification. The nerve ganglia of the entire fragment were counted for each serial cut, and the mean was obtained. The slides were scanned using a common printer to measure each fragment. Using the ImageJ software, the average of the four cuts was normalized to 1 cm, and the result was obtained as the number of ganglia/cm of the intestine.

Slides stained with picrosirius and hematoxylin were used for morphometric evaluation of the deposition of the connective tissue in the mucosa, submucosa, and intestinal muscle layers. For each intestinal fragment, 20 fields were analyzed at ×400 magnification following the methodology established in a previous study [18]. The results are expressed as the percentage of the collagen/animal.

### 2.4. Immunological Evaluations

The colon proximal fragment (approximately 1 cm) was transferred to an Eppendorf tube containing ×1 phosphate buffered saline solution and Complete™ protease inhibitor (Sigma, USA). The fragments were then homogenized in a homogenizer (DREMEL, EUA). The homogenates obtained were centrifuged at 12000 × g for 30 min, and the supernatants were stored at -80°C for quantification of cytokines and total proteins. The quantification of interferon gamma (IFN-*γ*) (R&D Systems), tumor necrosis factor-alpha (TNF-*α*) (R&D Systems), and interleukin- (IL-) 10 (BD OptEIA™) was performed on homogenates of the proximal portion of the colon using an immunoenzymatic assay (ELISA) according to the manufacturers' instructions. Tetramethylbenzidine (TMB) (3,3,5,5-tetramethylbenzidine) was used for the colorimetric reaction, and the optical density was measured using a microplate reader (Bio-Rad 2550 READER EIA, USA). To normalize the concentration of cytokines, they were used as total proteins of the modern intestinal homogenate in a nanodrop (Thermo Fisher Scientific, USA). The results are expressed in pg/mg.

### 2.5. Statistical Analysis

Statistical analyses were performed using the GraphPad Prism 8.0.1 (Graphpad Software, USA). The normality of the distribution of the quantitative variables was verified using the Shapiro-Wilk test. For comparison of the two groups, the Mann–Whitney test for data with nonnormal distribution was used. Results such as animal weight were analyzed using a two-way analysis of variance (ANOVA) test. For correlation, the Spearman's test was used. The results were considered statistically significant at *p* < 0.05.

## 3. Results

**Figure 1:**
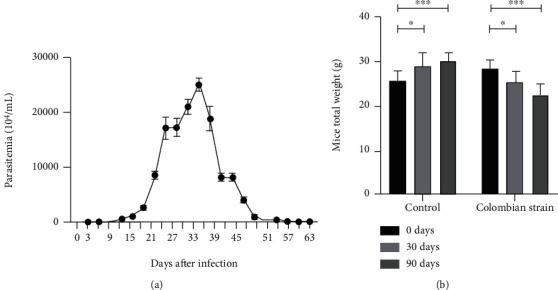
Blood parasitemia (a) and weight (b) differences between the acute and chronic phases of *T. cruzi* Colombian strain infected C57Bl/6 mice. Two-way ANOVA test. ^∗^Significant statistical differences at *p* < 0.05.

The count of circulating parasites showed slow transit parasitemia (Figure 1(a)). It started at 9 days after infection and declined completely on the 60th day. A peak was observed on the 33rd day after infection. At 30 days of infection (Figure 1(b)), there was a significant reduction in weight when compared to the day of the inoculum (*p* = 0.0192), which continued progressively until 90 days (*p* < 0.0001). Animals without infection gradually gained weight (0 days compared to 30 days, *p* = 0.0205, and 90 days, *p* = 0.0002).

**Figure 2:**
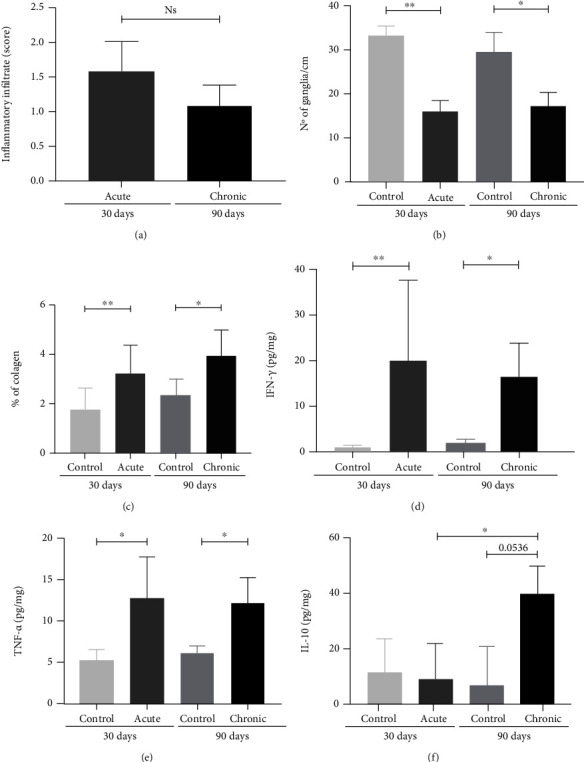
Intestinal immune and histopathological differences between the acute and chronic phases of *T. cruzi* Colombian strain infected C57Bl/6 mice. (a) Intensity of the intestinal inflammatory infiltrate. (b) Number of intestinal nerve ganglia. (c) Percentage of intestinal collagen deposition. Quantification of intestinal levels of (d) IFN-*γ*, (e) TNF-*α*, and (f) IL-10. Mann–Whitney test. ^∗^Significant statistical differences at *p* < 0.05.

**Figure 3:**
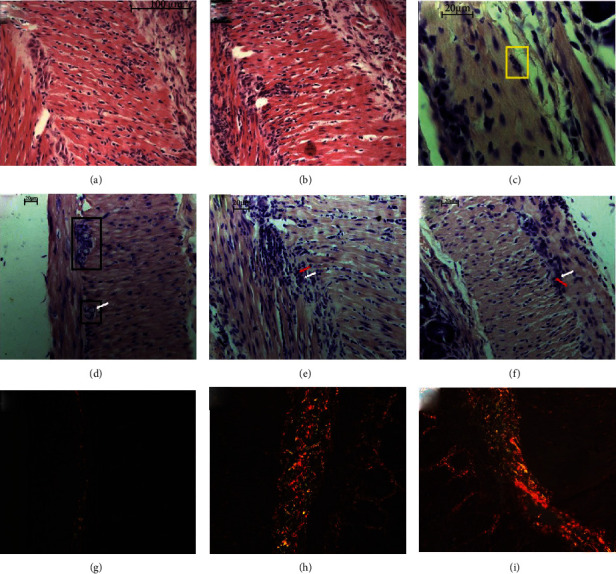
Intestinal photomicrographs of the intestinal histopathological differences between the noninfected, acute, and early chronic phases of *T. cruzi* Colombian strain infected C57Bl/6 mice. Intestinal inflammatory infiltrate (HE): (a) acute phase and (b) early chronic phase. (c) Intestinal amastigote nest in the acute phase highlighted by yellow lines (GIEMSA). Intestinal nerve ganglia (GIEMSA): (d) the two intestinal nerve ganglia of the myenteric plexus of uninfected mice highlighted using black lines (30 days); nervous ganglion without borderline of infected mice in the (e) acute and (f) early chronic phase with neuron (white arrow) and inflammatory cells (red arrow) remarkably close. Intestinal collagen deposition (Picrosirius): (g) noninfected, (h) acute, and (i) early chronic phase of infection.

To analyze the effects during the acute and early chronic infection, histological evaluations were performed. Regarding the presence of the inflammatory infiltrate (Figure 2(a)), during the acute phase, 100% of the animals showed moderate inflammatory infiltrate (1.30–2.06) (Figure 3(a)). During the early chronic phase, 50% of the animals are characterized by mild inflammatory infiltrate in the intestine (0.75-1) and the other half as moderate (1.15–1.46) (Figure 3(b)). Although there was a reduction, there was no difference between the experimental times (*p* = 0.1905) (Figure 2(a)). Although the quantification of amastigote nests is not performed, only one amastigote nest was found in a mouse during the acute stage of infection (Figure 3(c)).

Regarding the myenteric ganglion nerve, the acute phase was a determinant of structure reduction, which was demonstrated by a significant decrease when compared to the respective uninfected group (*p* = 0.0079) (Figure 2(b)). The same was observed when comparing the early chronic phase with its respective control (*p* = 0.0159). However, 90 days of infection were not enough to continue with the destruction of the ganglia when compared to that found in the acute phase (*p* = 0.9048). In the control group, preserved myenteric plexus architecture was observed (Figure 3(d)), both in the acute and chronic phases. Disorganization and intrusion of inflammatory cells close to the neurons of this structure were observed (Figures 3(e) and 3(f), respectively).

The collagen deposition process started in the acute phase and continued until the early chronic phase, which is demonstrated by the difference between the respective controls (*p* = 0.0303 and *p* = 0.0242, respectively) (Figure 2(c)). However, 90 days of infection were no longer sufficient to increase the collagen deposition compared to 30 days (*p* = 0.3524). Although uninfected mice had little intestinal collagen (Figure 3(g)), there was a great predominance of collagen fiber deposition in the intestinal submucosal layer in the infected mice at the two experimental times (Figures 3(h) and 3(i)). However, deposition in the mucosa and muscles was also observed.

Cytokines were used during both time points to analyze the intestinal immunological response. IFN-*γ* (Figure 2(d)) and TNF-*α* (Figure 2(e)), proinflammatory cytokines, were upregulated during the acute phase when compared with the respective controls (*p* = 0.0317 and *p* = 0.0079, respectively). In addition, the levels of TNF-*α* and IFN-*γ* remained high during the early chronic phase, without difference with acute levels (*p* > 0.9999 and *p* = 0.7857, respectively), but with differences compared to the respective control (*p* = 0.0357 and *p* = 0.0357, respectively). IL-10 (Figure 2(f)), an anti-inflammatory cytokine, was upregulated only during the early chronic phase when compared to the acute phase (*p* = 0.0357) and showed a tendency with the respective control (*p* = 0.0536), which suggests an attempt to control the immune response.

**Figure 4:**
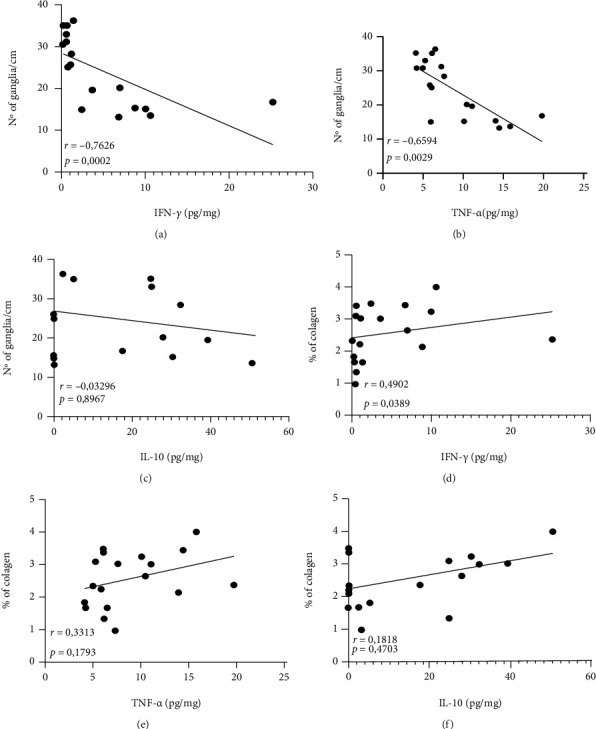
Correlations between the amount of nerve ganglia in the myenteric plexus with levels of intestinal (a) IFN-*γ*, (b) TNF-*α*, and (c) IL-10. Correlations between the percentage of intestinal collagen deposition with levels of intestinal (d) IFN-*γ*, (e) TNF-*α*, and (f) IL-10. Data obtained from uninfected animals in the acute phase and in the early chronic phase. Correlations were performed using the Spearman test. Significant statistical differences at *p* < 0.05.

After observing that both in the acute and early chronic phases, there were histological changes (increased inflammatory infiltrate, neuronal destruction, and collagen deposition) and the maintenance of a proinflammatory profile (IFN-*γ* and TNF-*α*) with an attempt to regulate (IL-10), and the next objective was to evaluate the relationship between these factors after 90 days of infection. Thus, it was observed that the increase in IFN-*γ* (Figure 4(a)) and TNF-*α* (Figure 4(b)) demonstrated a significant and negative correlation with the decrease in the nerve ganglia in the myenteric plexus (*r* = −0.7626 and *p* = 0.0002 and *r* = −0.6594 and *p* = 0.0029, respectively). However, IL-10 (Figure 4(c)) did not correlate with this decrease (*r* = −0.03296 and *p* = 0.8967). In addition, only the increase in IFN-*γ* (Figure 4(d)) correlated significantly and positively with the increase in intestinal collagen deposition (*r* = 0.4902 and *p* = 0.0389), while TNF-*α* (Figure 4(e)) and IL-10 (Figure 4(f)) showed no significant correlation (*r* = 0.3313 and *p* = 0.1793 and *r* = 0.1818 and *p* = 0.4703, respectively).

## 4. Discussion

The focus of this study was to evaluate whether the Colombian strain was capable of inducing immunopathological changes in the intestines of C57Bl/6 mice during the acute and early chronic experimental phases. Our results show that during the acute stage, there are intestinal changes, such as increased inflammatory infiltrate, neuronal destruction, and collagen deposition, along with the maintenance of the inflammatory process with proinflammatory cytokines until the early chronic phase.

A participant in DTU I and representative of biodema III, the Colombian strain, is defined by its low proliferative capacity, maximum peak close to 30 days, and myotropism [22, 23]. In addition, *T. cruzi* infection is characterized by excessive weight loss in experimental models, mainly in mice. These findings corroborate those of our model. This weight loss may be related to the inflammatory process, especially the presence of circulating proinflammatory cytokines such as TNF-*α* and IFN-*γ*, which may be associated with cachexia in experimental models [24, 25].

Infection with the Colombian strain and other strains of *T. cruzi* induces the appearance of the inflammatory intestinal infiltrate [4, 22, 26, 27], which also corroborates the findings of our model. However, the intensity of infiltration in the intestine may vary depending on the strain and inoculum concentration used in the infection [4, 9, 28]. It has been reported that infection by strain Y, for example, does not maintain the inflammatory process until the chronic phase, unlike what is observed in the Colombian strain [4]. This suggests that infection with the Colombian strain is more intense and stays longer.

Phenotypically, the inflammatory infiltrate in the human chagasic megacolon presents a great number of mononuclear cells, especially CD3^+^ lymphocytes [29]. In addition, eosinophils, mast cells, macrophages (CD68^+^), natural killer cells (CD57^+^), and cytotoxic T lymphocytes (TIA-1^+^) have also been reported in the organs of these individuals [30, 31]. The presence of these cells and the maintenance of the inflammatory process are associated with neuronal destruction, intestinal remodeling, and progression of chagasic megacolon and megaesophagus [32]. In addition, cells present in the enteric nervous system, such as the enteric glial cells, have also been associated with the progression of CD [31]. However, the role of these cells needs to be better understood. From this diverse cellular microenvironment, proinflammatory and regulatory cytokines, and microbicide components, such as nitric oxide (NO) and reactive oxygen species (ROS) can be produced by different cell types and induce neuronal death [32]. Although the characterization of these cells was not carried out in our study, it was demonstrated that the inflammatory infiltrate and proinflammatory cytokines persisted during the acute to the chronic phase, and this was correlated with neuronal destruction.

In addition, the maintenance of the intensity of the intestinal inflammatory infiltrate found in our study, which may be related to the production of proinflammatory cytokines (IFN-*γ* and TNF-*α*) were also maintained until the early chronic phase. IFN-*γ* is one of the cytokines most closely involved in resistance to *T. cruzi* infection [33–35], participating in the inhibition of intracellular replication of parasites [35], activation and maintenance of the T helper (Th) 1 response profile, and production of antibodies [36]. The inhibition of this cytokine in infected mice, for example, influences the increase in parasitemia, decreased survival, and decreased NO production [34]. Synergistically to IFN-*γ*, TNF-*α* activates macrophages with a microbicidal profile and results in the destruction of intracellular forms of the parasite; thus, it acts in the control of infection [37].

In the case of IL-10, a regulatory cytokine, only during the early chronic phase, there was an increase in the intestine. Differential production of IL-10 is one of the parameters that allow the differentiation of strains after infection [38]. A study that used a clone of the Colombian strain, Col cl1.7, demonstrated that this strain induced greater production of IL-10 compared to the infection established by strain Y in monocytes in vitro [38]. In an experimental model of the chronic phase, it has also been demonstrated that the Y strain does not induce changes in the production of this cytokine in the intestine of animals [10]. Thus, it is suggested that the intestinal increase observed in our study is related to a compensatory mechanism for controlling tissue damage due to the intense inflammatory process induced by the Colombian strain, which has also been suggested in experimental chagasic heart disease [20].

However, the intense inflammatory process established in the intestine is also related to tissue damage, especially the neuronal destruction [12, 39]. Arantes et al. (2004), using C57Bl/6 knockout mice for iNOS and IFN-*γ* infected with 100 blood trypomastigote forms of strain Y, demonstrated that the absence of NO was a determinant for neuronal survival after 10 days of infection. The failure to induce NO production via IFN-*γ* prevented denervation via oxidative stress in an experimental acute phase model [12]. This finding may explain the negative correlation between TNF-*α* and IFN-*γ* and the amount of the nerve ganglia in the myenteric plexus found in our study. Thus, the more the proinflammatory cytokines, the more is the NO, and the fewer are the neurons in the colon.

In addition to the participation of TNF-*α*, IFN-*γ*, and NO, other mechanisms related to the neuronal destruction in CD have been proposed. Substance *P*, a neuropeptide, has been shown to be increased in dilated portions of patients with chagasic megacolon, and this increase has been shown to be related to the induction/maintenance of intestinal inflammation and leukocyte chemotaxis, which may be related to neuronal damage [40, 41]. In addition, proteases produced by mast cells, such as tryptase, are correlated with neuronal death in patients with chagasic megacolon, mainly by decreasing immunoreactive PAR2 neurons [42]. Our group demonstrated that type 2 bone morphogenetic proteins are correlated with neuronal destruction and with the maintenance of the intestinal proinflammatory profile in an acute-phase experimental model infected with the Y strain [10].

Consequently, the positive correlation of IFN-*γ* with collagen deposition may also be related, since fibrosis proceeds the destruction of tissue. Contrary to what has been previously found for Y strain, which only included an increase in the connective tissue during the chronic phase of experimental infection [5, 10], our study demonstrated that the process of the collagen fiber deposition begins even in the acute phase of infection with the Colombian strain. What can also be related to the connected events of establishment of the inflammatory process with production of proinflammatory cytokines, destruction of neurons, and deposition of collagen fibers.

From these results, it is clear that the Colombian strain can also be used in experimental models to study the intestinal form of CD. The results of this study contribute to the understanding of the mechanisms related to the formation and progression of Chagas megacolon.

## Data Availability

All the data used to support the findings of this study are included within the article, figures, and references.
